# National Development and Regional Differences in eHealth Maturity in Finnish Public Health Care: Survey Study

**DOI:** 10.2196/35612

**Published:** 2022-08-12

**Authors:** Jari Haverinen, Niina Keränen, Timo Tuovinen, Ronja Ruotanen, Jarmo Reponen

**Affiliations:** 1 FinnTelemedicum, Research Unit of Medical Imaging, Physics and Technology Faculty of Medicine University of Oulu Oulu Finland; 2 Finnish Coordinating Center for Health Technology Assessment Oulu University Hospital Oulu Finland; 3 Medical Research Center Oulu Oulu University Hospital and University of Oulu Oulu Finland

**Keywords:** eHealth, electronic health records, picture archiving and communication systems, health information exchange, electronic prescribing, referral and consultation, videoconferencing, clinical decision support systems, health informatics, clinical informatics

## Abstract

**Background:**

eHealth increasingly affects the delivery of health care around the world and the quest for more efficient health systems. In Finland, the development of eHealth maturity has been systematically studied since 2003, through surveys conducted every 3 years. It has also been monitored in several international studies. The indicators used in these studies examined the availability of the electronic patient record, picture archiving and communication system, health information exchange, and other key eHealth functionalities.

**Objective:**

The first aim is to study the national development in the maturity level of eHealth in primary health care and specialized care between 2011 and 2020 in Finland. The second aim is to clarify the regional differences in the maturity level of eHealth among Finnish hospital districts in 2020.

**Methods:**

Data for this study were collected in 2011, 2014, 2017, and 2020, using web-based questionnaires from the *Use of information and communication technology surveys in Finnish health care* project. In total, 16 indicators were selected to describe the status of eHealth, and they were based on international eHealth studies and Finnish eHealth surveys in 3 areas: applications, regional integration, and data security and information and communications technology skills. The indicators remain the same in all the study years; therefore, the results are comparable.

**Results:**

All the specialized care organizations (21/21, 100%) in 2011, 2014, 2017, and 2020 participated in the study. The response rate among primary health care organizations was 86.3% (139/161) in 2011, 88.2% (135/153) in 2014, 85.8% (121/141) in 2017, and 95.6% (130/136) in 2020. At the national level, the biggest developments in eHealth maturity occurred between 2011 and 2014. The development has since continued, and some indicators have been saturated. Primary health care lags behind specialized care organizations, as measured by all the indicators and throughout the period under review. Regionally, there are differences among different types of organizations.

**Conclusions:**

eHealth maturity has steadily progressed in Finland nationally, and its implementation has also been promoted through various national strategies and legislative changes. Some eHealth indicators have already been saturated and achieved an intensity of use rate of 100%. However, the scope for development remains, especially in primary health care. As Finland has long been a pioneer in the digitalization of health care, the results of this study show that the functionalities of eHealth will be adopted in stages, and deployment will take time; therefore, national eHealth strategies and legislative changes need to be implemented in a timely manner. The comprehensive sample size used in this study allows a regional comparison in the country, compared with previous country-specific international studies.

## Introduction

### Background

The World Health Organization (WHO) defines eHealth as “the cost-effective and secure use of information and communications technologies (ICTs) in support of health and health-related fields, including health care services, health surveillance, health literature, and health education, knowledge and research” [1]. According to the WHO, eHealth has a clear and growing impact on the delivery of health care around the world and making health systems more efficient [1]. However, the use of ICTs in health care requires strategic and comprehensive national action to make the best use of it [1,2]. In practice, the term *eHealth* includes a wide range of applications from electronic patient record (EPR) to e–appointment booking (e-booking); therefore, eHealth maturity defines how these different applications were adopted [2-12].

The level of maturity and development of eHealth in health care has been monitored in several international studies [2,3,5-12]. One of the first comprehensive studies was published in 2011. It provided comparative information on the maturity level of eHealth in various European countries [3]. New study reports on eHealth maturity in health care have since been produced by the European Commission (EC), WHO, and Organization for Economic Co-operation and Development (OECD) [2,5,6,8-13]. The Nordic eHealth Research Network has produced comparative information on eHealth maturity levels in various Nordic countries [4,14-16]. In Finland, the development of health care organizations’ digitalization has been systematically studied since 2003, through surveys conducted every 3 years [17-21]. The most recent study was conducted in 2021 as part of the *Monitoring and Evaluation of Social and Health Care Information System Services* project [20,22]. It described the situation of digitalization in Finnish health care organizations in 2020 [20]. Although studies have mainly focused on the eHealth maturity level of health care service organizations, eHealth services provided for citizens have also been the subject of research, both in Finland and internationally [19-21,23,24].

Deloitte 2011 report included the EPR, picture archiving and communication system (PACS), e-prescribing, e-referral, e-booking, and telemonitoring (the possibility to use patients’ own health data) as the main applications describing the state of health care digitalization [3]. Moreover, the report examined how different countries implemented the wireless use of EPR [3]. EC studies also highlighted the abovementioned applications and the clinical decision support systems (CDSSs) as key indicators of eHealth maturity [5,10]. The degree of integration of CDSS can vary from a separate database to integration with the existing EPR, and Finnish follow-up studies have examined CDSS from this perspective [17-20].

According to a Deloitte report and an EC study, health care integration can be described as an organization’s relationship with external service providers, such as other hospitals and health care organizations [3,9]. In the EC study, the sharing of clinical care information, laboratory results, and radiology results between organizations was chosen as the key indicator of integration [5]. They also played an important role in the Finnish health care system, where it is still the case that different organizations largely produce specialized care and primary health care [17-21]. In Finland, Kanta health information exchange (HIE) services are being used from 2010 [25,26]. Although all public health care organizations have now joined Kanta, much of the information exchange continues to use regional HIE (RHIE) systems [18-21,27,28].

Strong user ID is one of the key ways of protecting a patient’s health information [10]. Therefore, e-ID and signature were chosen as one of the key indicators for describing data security in Finnish eHealth surveys [17-21]. There must also be sufficient personnel with computer skills to ensure data security practices [17-21]. Technical support for EPR users was chosen as an indicator to describe the reliability of EPR systems [17-21].

General tax revenues collected by the municipalities are the main source of funding for health care and social services in Finland [29]. The state also participates in the costs by paying a general, nonearmarked subsidy to the municipalities [29]. In Finland, municipalities have the primary legal responsibility to organize social and health care services for their residents [29]. Municipalities are responsible for organizing primary health care services for their residents and ensuring that its residents receive the necessary specialized care services [29]. Finland is divided into 21 hospital districts for the provision of specialized care [29]. Every municipality belongs to one of the hospital districts [29]. Decentralized responsibility for organizing health care services has created regional differences in the provision and availability of services [30]. The biggest change in Finnish health care services is the health and social services reform, which will enter into force in 2023 and shift the responsibility for organizing health and social services and rescue services from the municipalities to the 21 new well-being services counties [30]. Some hospital districts have already consolidated their services into a large entity, and in these organizations, specialized care and primary health care fall under the same administrative organization [18-20,31]. The aim of the health and social services reform is to provide equal services to citizens and further develop health care and its operating methods through digitalization [30]. Although the number of EPRs has decreased over the years in both specialized care and primary health care, one of the goals of the health and social services reform is to move toward common solutions for the procurement of EPRs [32,33].

The digitalization of health care has progressed well in Finland [17-21,24-28,33]. This has also come to the fore in international studies, which have highlighted the fact that Finland is one of the pioneers in the digitalization of health care [2,3]. Various national strategies and legal changes have also promoted the implementation of digitalization in Finnish health care [17-21,25,26]. This study aimed to provide information about eHealth maturity from the perspective of national development and regional differences. The data from this study can be used to examine how eHealth maturity has progressed nationally and in different hospital districts before the health and social services reform [30]. As the digitalization of health care has long been ongoing in Finland, the results can also be exploited internationally. The results show which application areas will be adopted first and how national strategies and legislative changes can contribute to the development of eHealth maturity, both nationally and regionally.

### Objectives

The main aims of the study were the following:

1. To study the national development in the maturity level of eHealth in primary health care and specialized care between 2011 and 2020
2. To clarify the regional differences in the maturity level of eHealth among hospital districts in 2020

## Methods

### Data Collection

This study used data collected in connection with the *Use of information and communication technology in health care 2020* survey and previous surveys in 2011, 2014, and 2017 [17-20]. The data for this study were collected from Finnish public health care providers. The target group for specialized care comprised all 21 hospital districts. In primary health care, the target group included all organizations specified as either independent municipalities or co-operation consortiums of municipalities with the responsibility to provide primary health care services.

The data for the surveys were collected during the first quarters of 2011, 2014, 2017, and 2020, using web-based questionnaires (Webropol; Webropol Ltd). The questions were kept comparable between the survey years. Medical directors and IT leaders (chief information officers) in specialized health care and chief physicians in primary health care were the survey respondents. The questionnaires were sent to them through email. The responses from the entire organizational level were compiled. In some hospital districts, specialized care is also responsible for the municipalities’ primary health care services. In these cases, the questionnaire was sent only for specialized care, and the responses regarding specialized care were transferred to the surveys for primary health care.

Table 1 presents the health care organizations that participated in the survey over different years. All specialized care organizations (21/21, 100%) responded to the questionnaire during the survey period. Municipal health care arrangement models changed during the survey years, creating variability in the number of primary health care organizations that participated in the survey and in the response rates and population coverage in different years.

**Table 1:** Health care organizations participating in the survey in different years.

Year	Respondents in specialized care (n=21), n (%)	Primary health care	
		Respondents, n (%)	Population coverage, %
2011	21 (100)	139 (86.3)^a^	91
2014	21 (100)	135 (88.2)^b^	95
2017	21 (100)	121 (85.8)^c^	95
2020	21 (100)	130 (95.6)^d^	99

^a^Sample size, n=161.

^b^Sample size, n=153.

^c^Sample size, n=141.

^d^Sample size, n=136.

### Indicators for eHealth and Their Analysis

In total, 16 indicators were selected to describe the status of eHealth (Table 2). They were based on the indicators in the eHealth report on specialized care, EC eHealth studies, and Finnish eHealth surveys in three areas: (1) applications, (2) regional integration, and (3) data security and ICT skills (Table 2) [3,5,10,17-21]. Traditionally, eHealth surveys have used the availability of applications or services as indicators [3]. However, availability saturation has been achieved in Finland in several health care application areas. For example, in 2010, EPR was available in all specialized care and primary health care organizations [17]. For several years, Finnish national eHealth surveys have also enquired about the intensity of use to describe the integration of an application or service into normal health care operations [17-21]. More specifically, it describes which proposition of a specific service is provided through eHealth means within an organization. For the intensity of use, the percentages (0%, 25%, 50%, 90%, 99%, and 100%) were chosen to correspond to the verbal answers, “not in use,” “a quarter,” “half,” “most,” “almost all,” and “all,” respectively. In the summary of indicators, the mean value of the results of the participating organizations was displayed. Where possible, the intensity of use of application was selected as an indicator to describe the use of eHealth. This better describes the deployment of eHealth in situations in which the functionality is already widely available.

**Table 2:** Indicators for eHealth maturity.

Areas and functionalities			Indicator		Responses
**Applications**					
	EPR^a^	Intensity of use		0=not in use, 2=≤25%, 4=≤50%, 7=≤90%, 9.9=≤99%, and 10=100%	
	Wireless use of EPR	Availability (local or external)		0=not available and 10=available	
	Picture archiving and communication system	Intensity of use		0=not in use, 2=≤25%, 4=≤50%, 7=≤90%, 9.9=≤99%, and 10=100%	
	Clinical decision support system	Integration level—average between the integration of diagnostic support and a drug interaction system		0=not available, 4=stand-alone web-based database on desktop, 6=database with access by navigating from the EPR, 8=automatic displayer of selected items, and 10=system for automatic integration of the EPR and database	
	e-Prescribing	Availability		0=not available and 10=available	
	e-Referral	Intensity of use		0=not in use, 2=≤25%, 4=≤50%, 7=≤90%, 9.9=≤99%, and 10=100%	
	Consultation e-referral	Intensity of use		0=not in use, 2=≤25%, 4=≤50%, 7=≤90%, 9.9=≤99%, and 10=100%	
	Teleconsultations via videoconferencing	Intensity of use—how often has the service been in use?		0=not in use, 4=less often, and 10=during the past 3 months	
	Possibility to use patients’ own health data	Availability		0=not available and 10=available	
	e–Appointment booking	Intensity of use—the patient selects an appointment time on their terminal (eg, computer) and it is transferred directly to the system		0=0% to 10=100%	
**Regional integration**					
	Exchange of clinical care information^b^	Availability		0=not available and 10=available	
	Exchange of laboratory results^b^	Availability		0=not available and 10=available	
	Exchange of radiology reports^b^	Availability		0=not available and 10=available	
**Information security and ICT^c^ skills**					
	e-ID and signature	Availability		0=not available and 10=available	
	Personnel with computer skills	Proportion		0=0% to 10=100%	
	Technical support for EPR	Intensity		0=not in use; 2=occasionally; 5=daily, but for less than normal office hours; 7=during normal office hours; and 10=at all times during the opening hours of the organization	

^a^EPR: electronic patient record.

^b^Health information exchange outside the centralized national Kanta services.

^c^ICT: information and communications technology.

### Ethical Consideration

The study followed the guidelines of the Finnish Advisory Board on Research Integrity [34]. The respondents were informed about the study, and they answered as representatives of the organizations being studied. No sensitive personal information was collected. The data were processed and stored in a secure environment, according to the procedures of the University of Oulu.

## Results

### Maturity of eHealth in Specialized Care and Primary Health Care Organizations at the National Level

#### Overview

**Figure 1:**
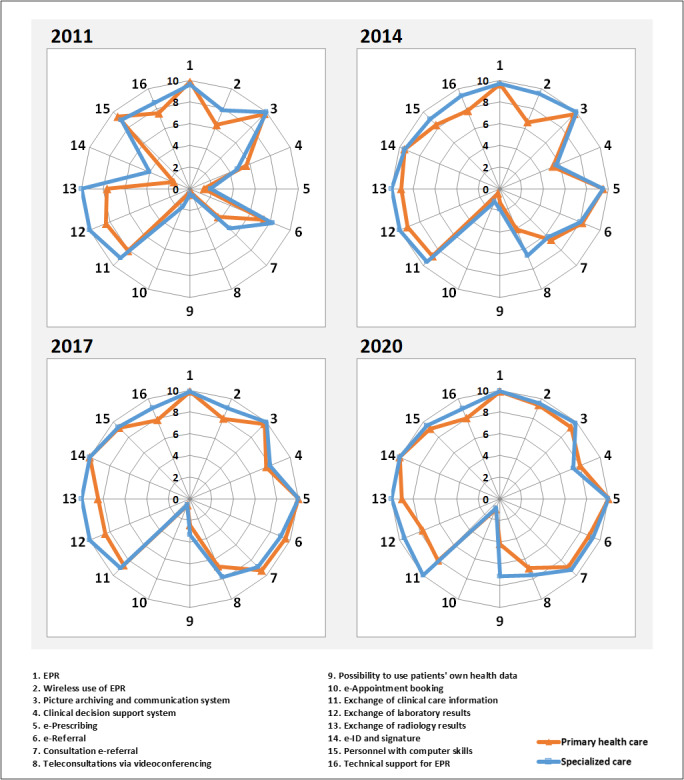
The national development in the maturity level of eHealth in the years 2011, 2014, 2017, and 2020 (modified from the studies by Reponen et al [19,20]). EPR: electronic patient record.

Figure 1 presents the national development in eHealth maturity in specialized care and primary health care organizations between 2010 and 2020. The results show that primary health care is generally behind specialized care organizations, as measured by all indicators and throughout the period under review. The biggest difference can be seen in the area of RHIE.

#### Applications

The EPR’s intensity of use has been at a high level since 2011, in both specialized care and primary health care organizations. There has been no significant change over the years. The wireless use of EPR has been available since 2014 in approximately all specialized care organizations (21/21, 100%), and there has been no significant change by 2020. In primary health care, availability has grown steadily, and in 2020, it has reached the same level as in specialized care. The intensity of use of PACS has been high in both specialized care organizations and primary health care centers in Finland since 2011. The integration level of the CDSS has increased since 2011; however, no growth can be seen after 2017. The level of integration has remained the same in both specialized care and primary health care organizations throughout the period under review.

The most significant change in the availability of e-prescribing occurred between 2011 and 2014. Since 2014, e-prescribing has been widely available in both specialized care organizations and primary health care centers. There was no significant change in the intensity of use of e-referral between 2011 and 2014. Since 2017, the intensity of use of e-referral has been high in both specialized care and primary health care organizations. The intensity of use of consultation e-referral increased between 2011 and 2017 in both specialized care and primary health care organizations. No significant change was seen by 2020.

In 2011, the intensity of use of teleconsultations via videoconferencing was very low, but significant growth was observed in both specialized care and primary health care organizations in the 2014 survey. In 2017, the intensity of use increased slightly, but remained at the same level in the 2020 survey. The possibility to use the patient’s own health data remained very limited in 2011 and 2014. There has since been significant growth in this area of application, especially in specialized care organizations. The intensity of use of e-booking remains low in the 2020 survey, and no growth can be seen throughout the survey period.

#### Regional Integration

All specialized care organizations reported that exchange of laboratory results and radiology reports was available from 2011. However, in 2020, not all specialized care organizations reported that regional information exchange of laboratory results was available. Exchange of clinical care information was unavailable in all specialized care organizations during the survey period, except in the 2020 survey, when all organizations (21/21, 100%) reported that it was available.

In 2020, approximately 80.1% (109/136) of the primary health care centers reported the availability of regional information exchange in all 3 areas of information exchange. There is no major change in the results of the 2020 survey compared with those of the 2010 survey. The highest reported availability of regional information exchange in primary health care centers was observed in the 2014 survey.

#### Data Security and ICT Skills

The availability of e-ID and signature was low in 2010, in both specialized care organizations and primary health care centers. In 2014, it was available in approximately all specialized care organizations (20/21, 95%) and primary health care centers (146/153, 95.4%), and it has been in use in all specialized care organizations (21/21, 100%) and primary health care centers (141/141, 100%) since 2017. Throughout the survey period, organizations have reported that the number of personnel with computer skills was approximately 90%. There are slight variations in the reported results among different years. EPR technical support at all times during the organization’s opening hours remains unavailable in some specialized care organizations (7/21, 33%) in 2020. No significant change can be seen in the results during the survey period.

### eHealth Profiles at the Regional Level

**Figure 2:**
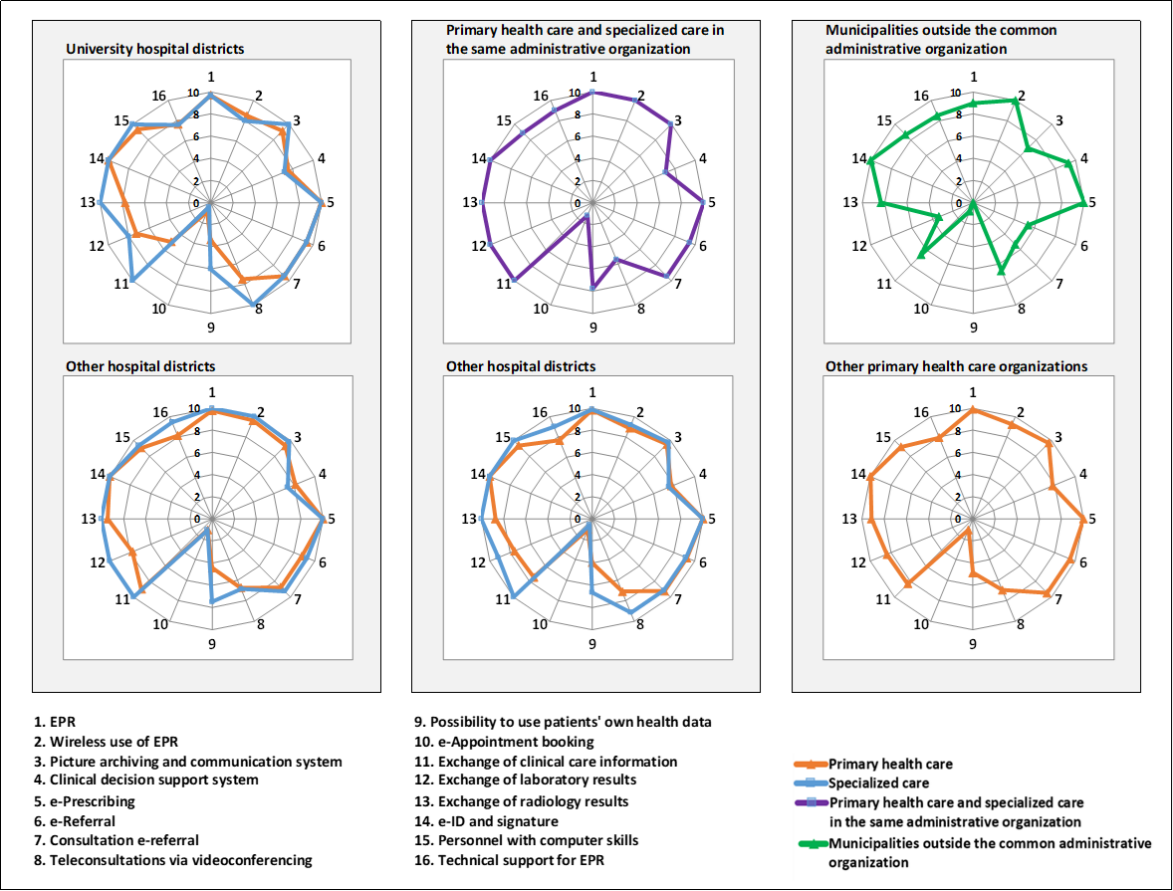
The status of the eHealth profiles of different types of health care organizations. EPR: electronic patient record.

The status of the eHealth profiles of different types of health care organizations is presented in Figure 2.

Especially in primary health care, the results do not show that the eHealth maturity level is better in large university hospital districts than in smaller hospital districts (Figure 2). For example, in university hospital districts, the availability of EPR technical support and wireless use of EPR is lower than the average of the other hospital districts. The availability of RHIE is also low in university hospital districts, especially for primary health care. Only the intensity of use of teleconsultations via videoconferencing is at a higher level than that in the university hospital districts.

In 43% (9/21) of the hospital districts, primary health care and specialized care are under the same administrative organization, and all these organizations use the same EPR brand throughout their municipalities and specialized care organizations (Figure 2). Compared with the other hospital districts, the combined organizations report better results for the availability of the wireless use of EPR and RHIE and the intensity of use of e-referral and consultation e-referral. The use rates of e-referral and consultation e-referral are low only in Kainuu. South and North Karelia still stand out from these organizations because of their good results. In both hospital districts, all indicators are saturated, except for the use of e-booking. In North Karelia, there is also scope for improvement in the number of personnel with computer skills.

In total, 14% (3/21) of the hospital districts also have individual municipalities outside the common administrative organization (Figure 2). In this 14% (3/21) cases, the results obtained from the municipalities outside the common organizations are worse than those at the national level. The results are particularly worse in the intensity of use of e-referral and consultation e-referral and the availability of laboratory result exchange. However, the integration level of the CDSS is higher in these municipalities than in the other primary health care organizations.

In addition to the 43% (9/21) of the hospital districts that have primary and secondary care under the same organization, 10% (2/21) of the districts reported that they were using the same EPR brand in both specialized care and primary health care organizations in their area, making a total of 52% (11/21). In all these organizations, the use rate of EPR was 100% and the wireless use of EPR was available. They reported better results in RHIE, especially when comparing with primary health care results. All these hospital districts, except Kainuu and Kanta-Häme, reported 100% use rates for e-referral and consultation e-referral. Of the 11 organizations, 8 (73%) organizations reported that EPR technical support was always available during their organization’s opening hours. Of the 10 hospital districts without the same EPR in use in their area, 6 (60%) districts reported that EPR technical support was always available during opening hours in specialized care organizations. Therefore, there was no significant difference in the results for specialized care organizations; however, in primary health care, the situation was different. None of the primary health care organizations without the unified EPR (104/136, 76.5%) reported that this service was always available during the organization’s opening hours.

The integration level of the CDSS varies greatly among hospital districts. In total, 14% (3/21) of the specialized care organizations reported that the CDSS was a stand-alone web-based database on the computer desktop, and 24% (5/21) reported that they had automatic integration of the EPR. Although the use of patients’ own health data significantly increased by 2020, regional differences in its availability still remained. Overall, 29% (6/21) of the specialized care organizations reported that this application was unavailable in 2020. The regional difference can also be seen in the intensity of use of teleconsultations via videoconferencing, because 19% (4/21) of the specialized care organizations reported that this application was not in use and the remaining 81% (17/21) reported that this service had been in use in the past 3 months. The intensity of use of e-booking remained low throughout the survey period, and no major regional differences can be seen in the 2020 survey results.

## Discussion

### Principal Findings

The digitalization of health care has progressed well in Finland, and its implementation has also been promoted through various strategies and legislative changes [17-21,25,26]. The progress of health care digitalization has also been systematically monitored through studies since 2003 [17-21]. The studies’ timing has aimed for alignment with the schedules of key legislative changes and strategies [17-21]. This survey study presented the development of eHealth maturity, measured by key indicators, in Finnish health care in 2011, 2014, 2017, and 2020. It also studied the regional differences in the maturity level of eHealth among hospital districts in 2020, measured by the same indicators. The study covered all Finnish specialized care organizations and a comprehensive portion of primary care organizations. In every study year, the response rate of primary care organizations was >86% and population coverage was >91%. The comprehensive sample size of this study’s respondents allowed regional comparison among organizations. Previous international studies have been based on sample data, so the results are presented country by country and not regionally within a country. For example, in the latest EC benchmarking study on the deployment of eHealth among general practitioners (GPs) in 2018, the sample size of Finland was 2.5% of GPs [8]. Internationally identified eHealth indicators were used in this study, and they remained the same between different study years; thus, the results were comparable. As the digitalization of health care in Finland has progressed, availability saturation has been achieved in some health care application areas [17-21]. In these cases, the intensity of use of the application was selected to describe the use of eHealth instead of availability [17-21].

Nationally, eHealth maturity has progressed steadily in Finland, with the biggest developments in eHealth maturity occurring between 2011 and 2014. On the basis of the national results, the functionalities have been built step by step. The first phase focused on key functionalities such as the deployment of EPR, PACS, and RHIE. Since 2011, the reported intensity of use of EPR has been high, and in the 2020 survey, only 5% (1/21) of the hospital districts reported the intensity of use of EPR as <100%. The intensity of use of PACS has been 100% in all hospital districts (21/21, 100%) since 2017. The next development step was to focus on the deployment of e-referral and consultation e-referral functionalities. Between 2011 and 2017, the use rate of these functionalities changed greatly, but the development has since stalled.

The uptake of the Finnish Kanta services started in 2010 [25]. Their introduction enabled the use of e-prescribing, which has since been widely adopted [26]. It became mandatory in 2017, which can also be seen in the result—since 2017, all specialized care organizations (21/21, 100%) and primary health care centers (141/141, 100%) have adopted it. The e-ID and signature also became mandatory with the introduction of the Kanta services, and since 2017, they have been introduced in all specialized care organizations (21/21, 100%) and primary health care centers (141/141, 100%). This emphasizes that changes in the law may lead to significant changes in eHealth maturity, as shown by a comparison of the results from 2011 to 2017 [25,26].

The development of smart devices and telecommunication networks has enabled the provision of an increasing number of remote and wireless services [3,19-21]. This is also reflected in the results because the availability of the wireless use of EPR and the use rate of teleconsultations via videoconferencing increased during the survey. The biggest development in these indicators can be seen between 2011 and 2014. The intensity of use of teleconsultations via videoconferencing has reached 80% since 2017, but no major improvement has been observable since then. Regionally, there are differences in the frequency at which this functionality has been used.

The aim of the health and social services reform is to improve the quality of services and ensure regional equality [30]. According to this study, there are still regional differences in the eHealth maturity levels among different hospital districts. There are differences in how comprehensive RHIE can be provided, how well EPR technical support is organized, and whether e-referral and consultation e-referral are widely used. These are particularly evident in the indicators for primary health care, especially in the municipalities outside the common administrative organization. Their results are noticeably worse in the intensity of use of e-referral, consultation e-referral, and RHIE. Regional variation also exists among hospital districts in how well CDSS is integrated into the EPR, the use rate of teleconsultations via videoconferencing, and the use of patients’ own health data. However, EPR and PACS are widely used in all specialized care organizations (21/21, 100%), and wireless use of EPR is available in most hospital districts (20/21, 95%). The results indicate that operating under the same administrative organization and using the same EPR brand in the region will enable the support of a more comprehensive level of eHealth maturity regionally for both specialized care and primary health care. On the basis of these results, it seems the goal of the health and social services reform to establish large operational units will improve the opportunities to provide a better and equal level of eHealth maturity [30]. The goal of the health and social services reform to move toward common solutions for the procurement of EPRs seems to help achieve better results in the intensity of use of EPR, availability of wireless use of the EPR, and availability of RHIE [32,33]. According to this study, a unified EPR brand also seems to allow slightly better EPR technical support.

Certain similarities can be observed if we examine the results in terms of how different hospital districts provide eHealth services for their citizens. This is especially true in the cases of South and North Karelia. Ruotanen et al [24] studied the availability of eHealth services for citizens in 2020. According to their study, these 2 hospital districts offer the widest range of eHealth services for citizens and they have the best eHealth maturity [24]. Could the explanation be that determined work has been done in these hospital districts to promote the digitalization of health care and implement national strategies? This may also be because organizations have had time to implement health care integration over a sufficiently long period because, for example, Eksote, the common administrative organization in South Karelia, has been operating since 2010 [31]. This highlights that an early investment in development is important, because moving functionalities into the production phase is a time-consuming process [7,25].

When this study’s results are examined internationally, we see that in the surveys conducted by the EC in 2013 and the WHO in 2016, Finland was ahead of the European Union (EU) and global average in the selected indicators [5,6]. For example, during those years, the availability of EPR and PACS was higher than the EU and global average, and even currently, the intensity of use rates for both applications in Finland are approximately 100% [5,6]. The biggest increase has been observed in the use of the patient’s own health data, because in the EC study in 2014, the use of the patient’s own health data was very limited at the EU level, as in Finland [5]. Moreover, in the Nordic study in 2014, the use of this functionality was low in all the Nordic countries. A Nordic benchmarking study noted that the Nordic countries were eHealth pioneers, especially in the HIE and EPR functionalities [4,5]. The Nordic eHealth Research Network also states in its study that several eHealth functionalities have already reached 100% availability in the Nordic countries; therefore, studies should focus more on the intensity of use of these functionalities [16]. Our study provides an example of how intensity of use data can be collected in a situation in which data on availability alone reveal insufficient details.

The latest benchmarking study results have shown that Finland remains as one of the pioneers in the development of eHealth. Ammenwerth et al [7] performed an international comparative study of 6 basic eHealth indicators across 14 countries in 2020. On the basis of their findings, Finland showed the best overall outcome in all the selected eHealth indicators in the study, followed by South Korea, Japan, and Sweden [7]. According to the study, Finnish health care professionals could easily access their patients’ health data and were able to add the data to electronic health records, but the possibility for patients to add data to their health records remains to be improved in Finland [7]. The 2017 OECD eHealth indicator survey, conducted in 38 countries, found that no country outperformed all countries in all the indicators used in the survey, but in contrast, no country lagged behind the other countries, as measured by all the indicators [35]. According to this study, Finland is one of the top performers in the availability of EPR and use of HIE of radiology results and images [35]. Finland was also noted as a top performer among OECD countries in technical and operational readiness to provide national health information from EPRs [12]. The availability to electronically request prescription renewal or refill and patients’ ability to access test results via the web was <50% in approximately all the OECD countries that participated in the 2017 study [35]. The availability of e-booking clearly needs to be improved among OECD countries, because in approximately all countries, including Finland, its availability was <50% [35]. The latest EC eHealth benchmarking study in all EU countries in 2018 highlighted Finland as one of the top performers, especially in the sharing of radiology test images and reports [8]. The EPR has been fully available across all EU countries since 2018. HIE is also well adopted in EU countries, but it has been less adopted than the EPR [8]. There was an increase in the adoption of HIE across all member states between 2013 and 2018, and along with Denmark, Estonia, and Sweden, Finland is among the top clinical data performers in HIE [8]. The highest HIE availability rates among EU countries were reported for receiving laboratory reports (77%), certifying sick leave (69%), sending and receiving referral and discharge letters (53%), and transferring prescriptions to pharmacists (52%) [8]. Compared with these results, this study shows that Finland is ahead of the EU average in the exchange of laboratory results, e-referral, and e-prescribing. Although a study conducted in 2018 found that e-prescribing was widely adopted in the 23 EU countries studied, there was great variation in authentication procedures among the countries [36]. One of the goals of EU for the development of eHealth has been to promote cross-border health care [37]. However, only Finland and Croatia have e-prescribing systems that can prescribe medications to be dispensed abroad [36].

Scope for development remains among EU countries, especially in the adoption of telehealth services and personal health records [8]. There is also scope for improvement among OECD countries in the adoption of telehealth services, because only approximately one-third of the hospitals indicated that they had telehealth capabilities for patient consultation [35]. In any case, consultation with other professionals using telehealth services is well adopted in Finland, because this study indicates that remote consultation via videoconferencing has been extensively adopted. However, there is still scope for improvement in Finland; for example, the use of CDSS was below the EU average in 2018 [8]. e-Booking in the Finnish public health care context clearly needs to be developed. On the basis of this study, no significant development has been seen during the entire 10-year follow-up period. However, 43% of EU GPs reported that their ICT systems allowed their patients to request appointments in 2018; therefore, Finland clearly has scope for improvement in this area [8].

This study was conducted in the Finnish health care environment, but we believe the findings are applicable to other countries that aim to develop health care further through digitalization. On the basis of the results, the deployment of eHealth applications will take time, and both legislative changes and national strategies may help to promote implementation [38]. According to the WHO, 58% of the countries that responded to their global survey in 2016 reported having an eHealth strategy [2]. In Finland, the first national strategy for applying ICT to health care and social welfare was introduced in 1995 by the Ministry of Social Affairs and Health [21]. Thus, Finland was one of the first countries, along with San Marino, Norway, and Canada, to have eHealth strategies or policies in place [2]. Strategies have since been used to promote the structured recording of patient data and the integration between systems and to increase the electronic exchange of information between patients and health care professionals [17-21,25,26]. Payne et al [39] studied the status of HIE among 6 countries, and they stated that the complexity of health care systems will present barriers to HIE. This is the case in Finland, because there are still regions where different organizations provide specialized care and primary health care and use the different EPR brands in their region. The study also noted that in countries that have successfully achieved HIE, the impetus came from the government [39]. In Finland, HIE between organizations has been promoted through the national Kanta services, in which all public health care organizations have joined [25,26]. Despite this national service uptake, which allows information exchange between organizations, there is still a possibility to use RHIE systems, as highlighted in this study [28]. The aim of the latest strategy is also to promote interoperable and modular architectures and information security and to ensure sufficient data connections [40]. Legal changes may also contribute significant improvements to eHealth maturity, as can be seen in this study’s results regarding the availability of electronic prescription and e-ID and signature. These functions became mandatory for all public health care organizations in 2017, and the results show significant development between 2011 and 2017 [21,25,26]. However, the implementation of the new functionalities will take time because the path of the Finnish national electronic prescription system from legislation to full implementation took 10 years [25]. A very important step forward to enable RHIE in hospital districts was a law that came into force in 2011, which allowed public health care to build common patient registries for hospital districts and primary health care organizations in each of the regions. After the law’s implementation, specific consent from a patient who is informed was no longer required for information retrieval [21].

The results reveal that a basic infrastructure such as the EPR must be in place to enable other advanced functionalities such as the CDSS and HIE, because the structured data storage of EPR is a prerequisite for the operation of CDSS systems [5,10,17-21]. Presumably, an EPR and broadband wireless infrastructure must be available for the wireless use of EPR [17-21]. The results also show that operating under a common regional administrative organization and using the same EPR brand will enable better overall eHealth maturity results, especially in RHIE, for both specialized care and primary health care, at least in the Finnish health care context. Although national strategies can guide the development of eHealth, the regions’ own determined work can also lead to even better results. The results highlight a few regions with high degree of eHealth maturity in the selected indicators in this study while providing comprehensive eHealth services to their citizens, as shown in the study by Ruotanen et al [24]. The organization’s own activities also affect the extent to which EPR technical support is provided and whether personnel’s ICT skills are promoted through training [2].

### Limitations

The results show that not all indicators may be relevant when examining the eHealth maturity of future public health care in Finland. When these eHealth maturity level studies started in Finland, most of the functionalities collected according to internationally used availability indicators were still in the development phase. However, some indicators, such as e-ID and signature and e-prescribing, have been saturated since 2017 and provided no additional information. Therefore, instead of using e-ID, a better indicator for the regional evaluation of data security could be the availability of a documented data security policy or data security plan.

Regarding primary health care, the number of survey respondents has decreased over time. This is explained by the merging of municipalities into large administrative entities. In contrast, the response rate of primary health care centers to the survey has increased during the survey’s implementation; thus, the sample size has differed slightly in the different survey years. This may cause minor variations in the results for different years.

The results of this study are based on the data provided by various organizations. In each organization, its management has compiled organization-specific responses from experts in different areas. Different experts may have responded to the survey in different years of the study; therefore, the questions may have been understood differently. However, efforts were made to assist the respondents by providing them with their responses from the previous survey year as a reference. The respondents may have represented the administrative organization; therefore, they may not have had a complete picture of the situation in practice. For example, the interpretation of the proportion of personnel with computer skills may vary among respondents. The interpretations of terms in various years may also vary, depending on what was topical at the time. The intensity of use of certain eHealth applications is based on respondents’ estimates rather than log data, meaning that there may be variation in results, depending on the respondent’s interpretation.

### Conclusions

eHealth maturity has steadily progressed nationally in Finland, and various national strategies and legislative changes have promoted its deployment. The biggest developments in eHealth maturity occurred between 2011 and 2014. Some indicators reached saturation and an intensity of use rate of 100%. However, the scope for development remains, especially in primary health care. Regionally, differences remain among different organizations. Some hospital districts have already been operating under a common administrative organization for a long time, and the results suggest that they will be more prepared for the approaching health and social services reform. The national eHealth strategies and legislative changes need to be implemented in a timely manner, because the results of this study show that the functionalities of eHealth will be adopted in stages and deployment will take time.

## Abbreviations

CDSS: clinical decision support system
e-booking: e–appointment booking
EC: European Commission
EPR: electronic patient record
EU: European Union
GP: general practitioner
HIE: health information exchange
ICT: information and communications technology
OECD: Organization for Economic Co-operation and Development
PACS: picture archiving and communication system
RHIE: regional health information exchange
WHO: World Health Organization
